# Unifying Environmental Stress Cracking and Mechano-Sorptive Creep Under the Umbrella of Mechano-Sorptive Phenomena

**DOI:** 10.3390/biomimetics11040276

**Published:** 2026-04-16

**Authors:** Yue Yan, Anil Misra, Paulette Spencer, Viraj Singh, Ranganathan Parthasarathy

**Affiliations:** 1 College of Engineering, Tennessee State University, Nashville, TN 37209, USA; yyan1@my.tnstate.edu; 2 Department of Civil and Environmental Engineering, Florida International University, Miami, FL 33199, USA; anmisra@fiu.edu; 3 Institute of Bioengineering Research, University of Kansas, Lawrence, KS 66045, USA; pspencer@ku.edu; 4 SLB, Houston, TX 77056, USA; virajbhushan@gmail.com

**Keywords:** mechano-sorptive phenomena, environmental stress cracking, mechano-sorptive creep, polymer thermodynamics, bound water, free water, irreversible deformation, hydrogen bond, constitutive variable

## Abstract

Mechano-sorptive phenomena (MSP) refer to the coupled mechanical response of polymers under simultaneous mechanical stress and fluid sorption. The most researched MSP are environmental stress cracking (ESC) and mechano-sorptive creep (MSC). ESC initiates at regions of localized stress and solvent sorption, presenting as brittle fracture, while MSC is characterized by large, time-dependent, and partially recoverable creep associated with transient bulk sorption. ESC experiments can however also result in significant plastic deformation, in which case the term environmental stress yielding (ESY) has been used. Similarly, MSC can evolve into tertiary creep followed by rupture, in which case the phenomenon is termed mechano-sorptive creep rupture (MSCR). Both behaviors originate from solvent diffusion into the amorphous phase, leading to disruption of non-covalent interactions between polymer chains. This review bridges seemingly disconnected research to illustrate that ESC and MSC represent extremes on a continuum of MSP, rather than disparate phenomena. We identify the principles of polymer thermodynamics and experimental methods necessary to separate polymer deformation under MSC into reversible stress-induced swelling and irreversible non-equilibrium deformation. Finally, we illustrate how MSP underline the functionality of several biomimetic materials including dentin adhesives, mutable collagenous tissue, spider silk, tendons, and articular cartilage, as well the synthesis of biomimetic materials by solvent vapor annealing assisted by soft shear.

## 1. Introduction

The term mechano-sorptive phenomena (MSP) is introduced as the mechanical response of a polymer to the simultaneous action of mechanical stress and interaction with an adsorbing or absorbing fluid. MSP can range from brittle cracking to ductile yielding with large reversible or irreversible deformation. The mechanical failure caused by the MSP depends on the effect of mechanical stress on the polymer–fluid interaction and the consequent proximity of the polymer’s state to its glass transition for the specified testing temperature, mechanical stress state, loading frequency, and fluid concentration. In the present review, the term MSP describes only the effects of physically active fluids in polymers. Chemically active fluids that cause irreversible chemical degradation of the polymer are not considered although they may be just as important, or more so [1], especially for biomedical materials and devices. Physical interaction between the polymer and the fluid involves dissociation of intermolecular non-covalent bonds in the polymer and manifests as bulk plasticization or reduced surface energy in the case of strong solvents. A distinctive feature of MSP is that the effect of the simultaneous action of mechanical stress and solvent ingress is greater than the sum of the effects of each factor in isolation [2].

MSP can encompass a wide spectrum of chemo-mechanical behaviors as evidenced by the extensive research on environmental stress cracking (ESC) [3,4] and mechano-sorptive creep (MSC) [5]. ESC is often described as the reduced time-to-failure of polymers in an aggressive agent by cracking or crazing at stresses much lower than the stresses that cause detectable damage in air. ESC is characterized by a reduced time-to-failure for a creep test performed on a notched sample in an aggressive agent as compared to the same test performed in air. An ESC failure typically happens with pseudo-brittle fracture involving fibrillation and crazing but lacking secondary and tertiary creep.

While large ductile deformations can be observed in ESC testing, the results are typically not classified as ESC, i.e., one research group has termed such phenomena environmental stress yielding (ESY) [6,7,8]. The phenomenon associated with ESY most closely resembles mechano-sorptive creep (MSC). The term ESY has been applied when the stresses cause large plastic deformation. In comparison, MSC shows features resembling secondary creep with recoverable deformation under sufficiently low stress magnitudes but progresses rapidly to tertiary creep and rupture when stress magnitudes are sufficiently large.

ESC and MSC are both MSP and as such share commonalities in terms of underlying mechanisms and experimental methods. Interestingly, despite the commonalities, research on these two phenomena has occurred in isolation. As a result, knowledge that was gained through experimentation and modeling of ESC and MSC has not been integrated. Integrating knowledge and understanding of the two phenomena offers significant promise for improving the predictability of material models for MSP. Potentially, MSP can be predicted solely from fundamental physical and chemical descriptors of the polymer and fluid [9].

There are several biological tissues that leverage MSP for stimuli-responsive behavior. One example is the orders of magnitude increase in storage modulus of mutable collagenous tissue (MCT) in marine organisms [10,11,12]. Although not spanning the glass transition, this phenomenon still involves simultaneous fluid exudation and increase in internal prestress, thus falling under the category of MSP. The supercontraction of spider silk is a quintessential example of MSP where the spider effectively locks the silk in its out-of-equilibrium glassy state using extrusion under tension, and exposure to moisture triggers immediate entropic contraction to a rubbery state [13,14,15,16,17]. The permanent deformation and eventual slackness clinically observed in human and equine dehydrated tendons is another classic example of MSP: when the tendons are loaded in an incompletely hydrated state, the plastic deformation resembles that observed in the irreversible part of MSC. All these natural applications of MSP are being harnessed for the development of biomimetic materials including artificial silk [18] with supercontraction for soft robotics and fluid-exuding artificial cartilage for mimicking human joint lubrication. Perhaps the most direct application of MSP is solvent vapor annealing with soft shear (SVASS), which is used to guide the self-assembly of polymers in the manufacture of biomimetic materials [19,20,21,22]. Although SVASS is not typically considered an MSP, the underlying mechanism is the synergistic enhancement of molecular mobility through simultaneous stress and sorption.

**Figure 1:**
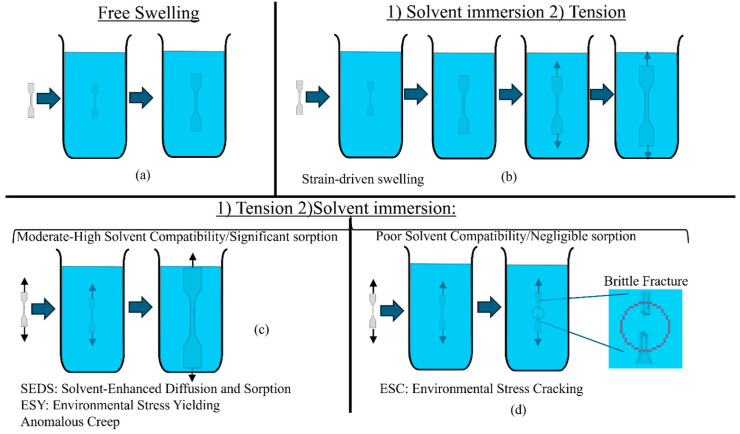
Schematic of four different types of mechano-sorptive processes classified by order of loading and macroscopic outcome: (**a**) free swelling, (**b**) strain-driven swelling, (**c**) solvent-enhanced diffusion and sorption/environmental stress yielding/anomalous creep, (**d**) environmental stress cracking.

This review provides a detailed examination of ESC, MSC and other MSP under one umbrella. The goal of this approach is to initiate the critical process of knowledge exchange across the phenomena. A schematic of four possible outcomes of an MSP on a crosslinked polymer is shown in Figure 1.

Figure 1a shows the well-known free swelling case, while Figure 1b shows the increased swelling and strain when a pre-saturated sample is subjected to tensile stress. Figure 1c shows the increased swelling and strain when a dry sample under tension is subjected to solvent saturation. Figure 1d shows an example of brittle fracture, which occurs when tensile stress is applied to a polymer in the presence of a poorly compatible solvent. All these cases have been covered in the existing literature under various terminologies, such as free swelling, strain-driven swelling, mechano-sorptive creep, and environmental stress cracking represented in Figure 1a, Figure 1b, Figure 1c and Figure 1d, respectively. The cases represented by Figure 1a, Figure 1b, Figure 1c and Figure 1d are referred to as case (a), (b), (c) and (d), respectively, in the remainder of the paper. Cases (a) and (b) are mostly relevant to applications where the polymer is initially saturated, e.g., hydrogels used as soft actuators, sensors, drug delivery systems, and numerous other applications. Cases (c) and (d) are more relevant to conditions where the polymer is initially in a dry state but may contact fluids in the application process. For example, thermoplastics in aerospace components exposed to paint strippers and jet fuels, cable jacketing and automotive parts exposed to cleaning agents or lubricants, timber structures exposed to moisture, and so forth. Material processes involving dentin adhesives and food grains may undergo MSP involving all cases (a) through (d). Although both ESC [23] and MSC [24] have been identified as potential failure mechanisms for dentin adhesives and other dental materials, they are not typically examined from a mechano-sorptive perspective. Therefore, this review is particularly relevant to the durability of polymeric dental materials, including composite restorative materials, from the perspective of MSPs. This review provides guidelines for such investigations.

## 2. Commonalities in the Molecular Phenomena Underlying ESC and MSC

The term mechano-sorptive creep (MSC) has been mostly used for the partially reversible creep of polymers under moisture cycling. It is characterized by a “molecular Velcro” mechanism where hydrogen bonds between polymer chains are disrupted by solvation and mechanical stress causes partially irreversible slippage between the corresponding polymer chains. Depending on the stress level, the hydrogen bonds may be partially or completely reformed after the removal of the stimulus, leading to augmented anomalous creep beyond that of fully saturated polymer samples. Under sufficiently high stress, MSC transitions into secondary and tertiary creep, leading to creep rupture [25,26,27]. MSC has been mostly studied for different types of woods and wood-based cellulosic polymers, and the solvent has generally been water or water vapor [28]. ESC, on the other hand, has been studied for a wide variety of amorphous and semicrystalline polymers including crosslinked polymers. Like MSC, ESC also involves the disruption of non-covalent interactions between polymer segments of the amorphous phase. In ESC, the non-covalent interactions are usually tie molecule entanglements in the case of semicrystalline polymers and hydrogen bonds in the case of amorphous polymers, while for MSC, they are often hydrogen bonds. In addition, ESC also reflects the effect of the fluid on the surface energy of the polymer.

The molecular-level mechanisms underlying both MSC and ESC, as with all MSP, are inherently time-dependent by virtue of the viscoelastic nature of the polymer as well as diffusion of the fluid molecules in the polymer. Both mechanisms happen primarily in the amorphous region of the polymer, which is accessible for fluid diffusion. The crystalline regions do not participate in any major way in MSP. For example, MSC as studied in wood and cellulose shows that only the non-crystalline portions are accessible to moisture [29,30]. Similarly, the underlying mechanism behind ESC is primarily polymer–fluid interaction in the amorphous regions and the consequent void formation followed by fibrillation, drawing and failure.

Despite resulting in visually different macroscopic failures, ESC and MSC overlap significantly. Under sufficiently high stress, MSC progresses to mechano-sorptive creep rupture (MSCR) [26,31,32]. ESC caused by moderately strong solvents interacting with amorphous polymers can induce a gel layer at the crack tip that prevents crack propagation [3,33], leading to a ductile failure, which is not unlike what is observed for MSCR [26]. Researchers have postulated that the brittle failure associated with ESC in amorphous polymers is caused by localized swelling due to non-solvents or weak solvents that induce tension in the material subjacent to the swollen gel layer. On the other hand, MSC and MSCR are observed for good to moderately good solvents due to bulk sorption [3]. Further details are provided in Section 3.3 and Section 4.

## 3. Evidence That ESC and MSC(R) Are Variants of the Same Underlying Phenomenon

**Figure 2:**
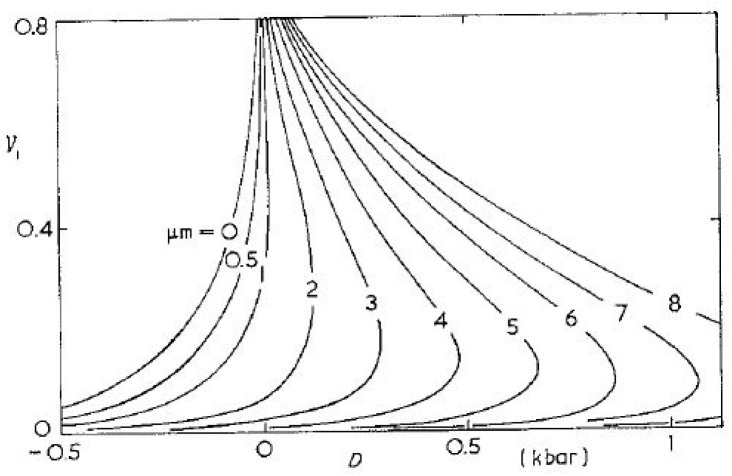
Equilibrium volume fraction of swelling liquid versus dilatant stress from the modified Flory–Huggins swelling relation reproduced from Gent [35].

The general equation for the chemical potential for a system at a constant pressure and temperature can be written as

(1) $$\mu ={\frac{\partial G}{\partial n}|}_{T,P}={\frac{\partial U}{\partial n}|}_{T,P}+{P\frac{\partial V}{\partial n}|}_{T,P}-{T\frac{\partial S}{\partial n}|}_{T,P}$$

where μ is the chemical potential, G is the Gibbs free energy, U is the internal energy, n is the number of moles of the solvent, V is the volume of the polymer–solvent system, T is the temperature of the system (held constant) and P is the external pressure on the system (held constant). Flory’s development of the thermodynamics of polymer–solvent interaction (see chapter XII of [34]) obtains the chemical potential difference between the polymer–solvent mixture and pure solvent for the case of free swelling under no external stress (P = 0 in Equation (1)). From Equation (1), it is straightforward to show that hydrostatic tension decreases the Gibbs free energy of the polymer–solvent system further below that of a stress-free condition, with reference to the pure solvent, while pressure increases it closer to that of the pure solvent. Gent [35] extended Flory’s approach to hydrostatic tension (negative P in Equation (1)), and the obtained relationship [35] is reproduced in Figure 2, where μ_m_ is the polymer–solvent interaction parameter (introduced by Flory [34]), ν_1_ is the equilibrium degree of swelling, and D is the dilatant stress (negative pressure). Small values of μ_m_ indicate a high polymer–solvent compatibility. From Gent’s relationship in Figure 2, compatible solvents cause significant swelling even with negative dilatant stress (positive pressure). Similarly, the poorer the polymer–solvent compatibility, the higher the dilatant stress required to cause significant swelling. Gent’s relationship shows the dramatic increase in sorption when hydrostatic tension is sufficiently high. Thus, Gent hypothesized that ESC in amorphous polymers was caused by increased swelling in zones of stress concentrations, which caused tensile stress in underlying regions still in the glassy state. Gent’s predictions have been corroborated by several researchers, e.g., Tynnyi [36] and Wolf [7,8,37].

### 3.1. Swelling and Diffusion Responses Driven by Chemical Potential Gradients

It is understood that the equilibrium between the swollen polymer and the solvent is achieved when the chemical potential inside the polymer–solvent system becomes equal to that of the pure solvent in the surrounding environment. The Smoluchowksi equation [38] fundamentally reveals that the rate of diffusion of the solvent into the polymer is driven by the gradient of the chemical potential across the polymer–solvent boundary. Therefore, from Equation (1), it follows that hydrostatic tension would cause an increase in swelling, and hydrostatic pressure would decrease swelling. Gent argued similarly and derived equations for uniaxial tension originally explored by Flory [39] and Gee [40] and for biaxial cases studied by Urayama [41] and others. Lejcuś and colleagues [42] reported that external loads (such as soil pressure) significantly suppressed the swelling rate and the final equilibrium swelling capacity. As the soil load increased, both the swelling rate and the equilibrium swelling capacity showed a clear decrease.

Using Equation (1), it also follows that the rate of solvent diffusion into the polymer should be greatly increased by the presence of hydrostatic tension since the diffusion rate is a function of the chemical potential gradient. This increase was observed for polyether ether ketone (PEEK) using several solvents. The increase in the rate of diffusion and solubility with uniaxial tension and residual stress was termed SEDS (stress-enhanced diffusion and solubility/swelling) by Wolf’s research group [6,7,8]. A similar increase in the rates of diffusion of kerosene and acetone in polytetrafluoroethylene (PTFE or Teflon) under uniaxial tension has been reported by Tynni’s research group [36]. Wolf and colleagues reported that the induction period for initial swelling via toluene into unstressed PEEK was five to six orders of magnitude larger than that into PEEK under uniaxial tension greater than 25 MPa [37]. The increase in diffusion rates under tension can also be understood in terms of Eyring’s theory, where bond dissociation and association rates are functions of the chain force [43,44]. The solubility data provided by Wolf agree with the theory proposed by Treloar [45,46]; Treloar’s theory is applicable to solubility, but it is not suitable for diffusion processes. There has been limited research on MSC and ESY for amorphous polymers. In distinct contrast, there has been an extensive investigation of MSC in wood and wood-related materials, and ESC for amorphous and semicrystalline polymers. Singh and colleagues [24] studied MSC/ESY in methacrylate-based polymers interacting with water. Toratti [26] has studied MSCR, which parallels ESY in wood. Fukumori and colleagues [47] used nuclear magnetic resonance (NMR) to show that tensile strain enhanced the increase in molecular mobility of an acrylonitrile–butadiene copolymer rubber matrix under carbon tetrachloride sorption [47].

For simple crosslinked networks, MSC has not, to the best of our knowledge, been explicitly studied through experiments. In several studies, MSC is treated as a separate strain component, distinct from swelling-induced strain and viscoelastic strain. However, as we elaborate in the following section, and as noted by Reichel and Kaliske [32], the reversible part of the anomalous strain observed under simultaneous tensile stress and solvent diffusion for sufficiently small stresses can be fully explained in the framework of gel thermodynamics. If all the anomalous strain is reversible, mechano-sorptive creep is not an independent strain mode but is inherently included in the swelling-induced strain. The orders-of-magnitude difference in diffusion rates between swollen gels and initially dry gels could be why MSC is mistakenly regarded as being entirely independent of swelling-induced strain. For materials like wood, which possess a complex hierarchical structure, the manifestation of MSP is more nuanced.

### 3.2. Path-Independence of Loading and Swelling

**Figure 3:**
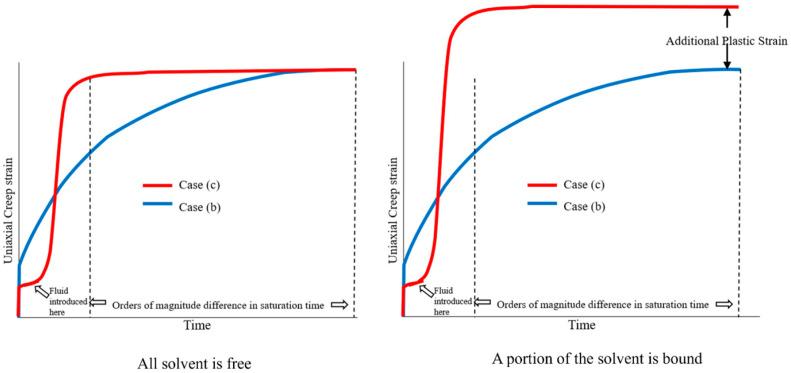
Schematic comparing creep strains in cases (b) and (c) for two scenarios: when all solvent is free and applied tensile stresses are sufficiently low to prevent yield, and when the bound solvent fraction is not negligible and/or applied tensile stresses cause yield and plastic strain.

The swelling behavior of polymers, including hydrogels, originates from a thermodynamic balance between osmotic pressure and the elastic restoring force of the polymer network. This understanding is rooted in the principle of thermodynamic equilibrium, where the final state is determined by minimizing the system’s free energy, regardless of the loading path. For a thermodynamically reversible process where the final equilibrium or steady state reached by a system should be independent of the order in which boundary conditions are applied, Gent’s hypothesis leads to the conclusion that a polymer will reach the same equilibrium condition for cases (b) and (c) from Figure 1b and Figure 1c. We note that the equilibrium thermodynamics-based Flory–Rehner theory considers all solvent molecules as free and does not account for the bound portion of solvent molecules. Therefore, if all the solvent is free and the stress is sufficiently low, cases (b) and (c) should reach the same equilibrium sorption and creep strain. However, when the bound solvent content is significant, case (c) is likely to lead to much higher creep strains and possibly greater equilibrium sorption as compared to case (b). This is because only the bound solvent, distinct from “bulk” free solvent, is irreversibly bonded to the polymer chains and is involved in irreversible deformation under simultaneous stress and sorption associated with MSC—all interactions with “bulk” free solvent are necessarily reversible as per polymer–solvent equilibrium thermodynamics. The sorption kinetics for case (c) are expected to be orders of magnitude faster than case (b) since a dry polymer creates a higher chemical potential gradient against pure solvent, as compared to an initially saturated sample. This difference makes it difficult to separate the deformation caused by non-equilibrium thermodynamic processes from a comparison of cases (b) and (c). However, the reversible component of the deformation from case (c) is expected to match case (b). A schematic of this argument is shown in Figure 3, which shows a comparison between cases (b) and (c) when all the solvent is free, and when a portion of the solvent is bound.

**Figure 4:**
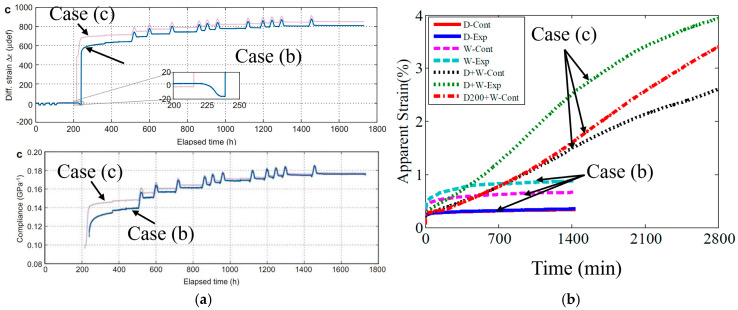
(**a**) Comparison in the evolution of creep compliance for samples started dry vs. started wet, reproduced from Montero [48], and (**b**) comparison in the creep strain for samples started dry vs. started wet, reproduced from Singh [50].

Experiments paralleling cases (b) and (c) can be found in the literature. For example, in the case of Norway spruce wood loaded in four-point bending, it was reported that after enough cycles, the response is independent of the humidity condition at initial loading. Montero and colleagues reported this observation after comparing initially dry and initially saturated specimens [48]. The compliance of the wet loaded samples tended to catch up with the response of the dry loaded samples. Furthermore, the responses of the initially dry and initially saturated samples were closely matched after several moisture cycles. Since bound water is expected to be effectively saturated within the first cycle, this observation indicates that the relatively small change in bound water in subsequent loading cycles leads to the convergence in the behavior of wet and dry loaded samples. This effect can be investigated further from the results of Singh et al. [24], who demonstrated a marked difference in the creep between stored dry-tested wet and stored wet-tested wet methacrylate-based dentin adhesive polymer samples. Even after accounting for the orders-of-magnitude difference in sorption kinetics between these samples, the difference in the results from the creep testing is likely driven, in part, by non-equilibrium thermodynamics. In partial support of this explanation, samples of this composition were found by Parthasarathy et al. [49] to have a relatively high content of bound water compared to polymers with a significantly reduced crosslink density. The classification of total water or solvent into free and bound components as well as the decomposition of MSC into reversible and irreversible parts is therefore an important step in understanding the nature of the MSC—this point has not been sufficiently appreciated in the literature. The results from Montero and Singh have been reproduced in Figure 4a and Figure 4b respectively. These observations suggest that the reversible part of MSC is explained by equilibrium theory of polymer swelling and does not require the rate of solvent sorption as a constitutive variable in the material model.

Further evidence is available from the work of Armstrong and Christensen [51] on MSC of eucalyptus and pine wood under bending, where two points are clear: (a) there is a significant recovery of the sample upon unloading under wet conditions, (b) MSC decreases with subsequent absorption cycles, and (c) final deflection of the sample when saturation was reached did not depend on the rate of sorption, but did depend on the gradient of vapor pressure. This indicates that a significant portion of the MSC that was reversible under wet conditions happened with respect to free water, especially with an increasing number of cycles, once the bound water was saturated.

**Figure 5:**
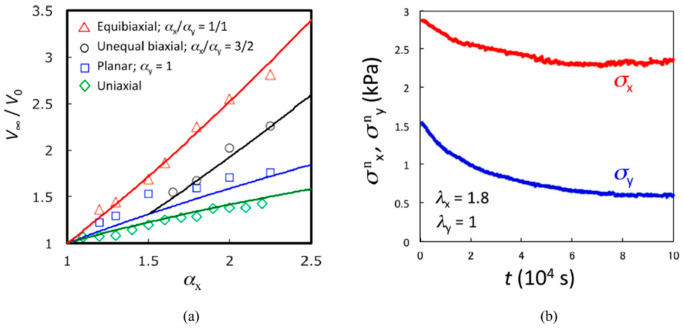
(**a**) Increased swelling under application of biaxial strain and (**b**) stress relaxation in the directions of biaxial strain application; figures reproduced from Fujine [41].

Future work is required to provide thorough, experimental validation of the arguments that are presented in this section. Experiments resembling case (b) have been performed for poly(acrylamide) gels [41,52] and rubber vulcanates [39,40,47], demonstrating increased swelling under tension. Similarly, numerous researchers [53,54,55,56,57,58,59] have thoroughly investigated the mechanical response of polymers under axial stretching in swollen states, and most of these studies were conducted under fully swollen unstressed equilibrium prior to deformation, i.e., corresponding to case (b). As an example, the results from Fujine [41] and colleagues have been reproduced in Figure 5a,b.

**Figure 6:**
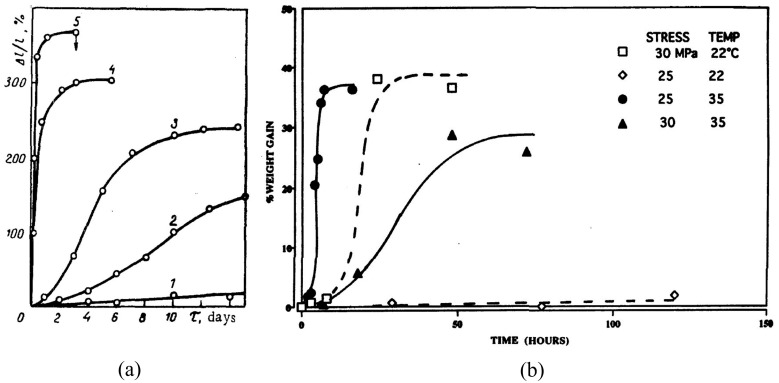
(**a**) Variation in creep strain of Teflon-3 in acetone under uniaxial stress, reproduced from Soshko [36], and (**b**) increasing sorption of toluene in 29% crystalline PEEK as a function of uniaxial stress and temperature, reproduced from Wolf [37].

The figures show that axial tensile strain on a hydrogel immersed in water causes increased swelling in the lateral direction and stress relaxation in the directions of strain application. Experiments like case (c) have been performed for a wide variety of polymers, e.g., references [7,8,37,60,61,62,63,64,65]. As examples, the results of Soshko [36] and Wolf [37] have been reproduced in Figure 6a,b.

They show how tensile stress greatly increases the equilibrium sorption and accelerates the sorption kinetics for (a) Teflon-3 in acetone [36], and (b) 29% crystalline PEEK in toluene [37], respectively. However, experimental results explicitly comparing polymers tested under cases (b) and (c) are limited and considerable work is required in this direction to better understand MSP. Given the similarity in causative stimuli, it is highly noteworthy that the above points are strongly relevant for ESC as well and need to be appropriately incorporated into any constitutive theories modeling ESC.

Irreversibility can also arise when stress-driven plastic yielding occurs for a particular solvent saturation. It is likely that the stress limit of linearity proposed by Reichel and Kaliske [31] is related to this yield stress. When the applied stress is greater than the yield stress, which depends on the solvent fraction and the temperature, the equilibrium deformation of case (c) is expected to exceed that of case (b). This observation is explained as follows: when the stress exceeds the yield stress under the conditions specified for case (c), there will be significant plastic deformation in the polymer, which reaches a maximum for conditions where the plasticization is sufficient for the glass transition temperature to drop below the testing temperature. This plastic deformation is termed pseudo-plasticity [66] since it is reversible with an increase in temperature. This plastic deformation adds to the tension-induced swelling, thereby increasing the overall deformation in case (c) as compared to case (b). It must be noted that in MSC experiments, the yield stress will not be a uniform value across the volume of the sample, but rather vary with the spatial distribution of the solvent. The extent of MSP is strongly dependent on the macroscopic sample size. The regions with greater amounts of solvent will be more plasticized and have a lower yield stress.

For conditions involving very low concentrations of solvent either due to low compatibility or low concentrations of a compatible solvent, there are two phenomena possible: (a) ESC or (b) antiplasticization. In the case of ESC, as described in Section 2 and in Figure 1d, the mechanism in amorphous polymers is primarily due to high tensile stress in regions underlying localized swelling, with reduced surface energy for fracture propagation also playing a role. Antiplasticization happens when at low concentrations, solvent molecules in a bound condition act as bridges between polymer chains, whereby some polymers experience increases in stiffness, glass transition temperature and tensile strength accompanied by lowered fracture strain since certain segmental motions of polymer chains could be restricted by these bridges [67]. With increasing solvent amounts, the transition to free solvent clusters and therefore “full plasticization” takes place. This transition has been observed and traced back to the presence of bound water for dentin adhesive polymers [49], although a feeble reduction in stiffness and strength, rather than clear antiplasticization, was observed prior to the onset of “full plasticization”.

### 3.3. Path Dependence in Sorption Isotherms

Sorption hysteresis is a phenomenon commonly observed in polymers, especially cellulose. The underlying mechanism behind hysteresis has been demonstrated by Chen et al. [68,69] to involve the combined effect of the pore structure and overall hydrogen bond network, leading to a complex energy landscape. The hysteresis shows that during desorption, the amount of adsorbed water is greater at the same relative humidity (RH) as compared to sorption. An inadequate equilibration time at each relative humidity value of the sorption–desorption experiment, particularly at low RH values where the polymer is below its glass transition temperature [70], could also be a contributing factor [71].

There are clear parallels between the order of stimulus dependence described in Section 3.2 and the path dependence observed in sorption–desorption hysteresis in that they both are governed by the kinetics and energetics of bound water formation. Chen et al. [68,69] demonstrated using a hybrid Monte Carlo and molecular dynamics simulation in cellulose that the formation of free water clusters begins once most hydroxyl sites on the polymer chains are already bonded with water molecules and unavailable for further bonding. Along these lines, it is highly likely that the presence of tensile stress makes previously unavailable sites accessible for hydrogen bonding with water. Section 3.2 explains the effect of stimulus order from the viewpoint of macroscopic thermodynamics, but a molecular-level explanation remains to be established. It is highly likely that the underlying mechanism for the path dependence described in Section 3.2 is also due to the complex interaction between the pore structure and hydrogen bond network involving polymer–water and water–water hydrogen bonds. The results at the macro scale however manifest differently because the bound water involving polymer–water hydrogen bonds is removed upon drying, but not upon the unloading of tensile stress. Thus, in the case of sorption–desorption hysteresis, only the kinetics and not the equilibrium state are affected by the direction of water transport, whereas in the case of MSC, both the kinetics as well as the final equilibrium state are affected by the order of stimulus. The plastic deformation because of the MSP described in Section 3.2 is thus irreversible with respect to mechanical load but is recoverable upon drying. The effects are also more significant because the initial condition is different (saturated versus dry).

This difference in reversibility between the sorption–desorption and MSC also necessitates different requirements for a constitutive model, which can capture MSC as compared to one that can capture the sorption–desorption hysteresis. While poromechanics constitutive laws incorporating the Langmuir adsorption model and its extensions [71,72] based on Maxwell relations incorporate the effect of applied stress on the sorption, they require reversibility to hold, which makes them applicable to model the effect of stress on sorption isotherms, but not for modeling the effect of sorption on the mechanical behavior. While the sorption–desorption process is reversible for both bound and free water with respect to drying, there is generally a component of creep strain generated during MSC, which is not reversible upon unloading. Also, the volumetric strain is not necessarily equal to the volume of adsorbed water during the swelling process of polymers.

### 3.4. Experimental Studies Spanning the ESC–MSC Transition

Despite the vast amount of literature on both ESC and MSC, there are very few investigations that demonstrate both ESC and MSC for the same polymer under different conditions of solvent compatibility, exposure time, mechanical stress and temperature. Such investigations are however crucial in qualitatively and quantitatively describing ESC and MSC as particular cases in a generalized spectrum of MSP. Rudakova [2,73] was one of the first authors to explicitly state that the simultaneous action of mechanical stress and the aggressive medium could lead to failure resembling either ESC or MSC. Results pertaining to this philosophy have been compiled in Table 1. Table 1 details conditions that led to either an ESC or an ESY/MSCR type of failure for the same polymer under different testing conditions.

It is apparent from Table 1 that for a given polymer, when the fluid only causes localized swelling, either due to low fluid solubility or insufficient diffusion times, a brittle failure associated with ESC results, while large deformation and ductile failure may result in an MSC or MSCR/ESY in the case of significant absorption. In the case of a compatible solvent, the behavior is controlled by either bulk plasticization or surface energy reduction [79]. Therefore, ESC and MSC can be unified under a single common umbrella of MSP, which can have far-reaching consequences in enriching both experimental and modeling studies of polymers, particularly in applications involving biomedical materials and devices.

## 4. Constitutive Models for MSP

Since the focus of this review paper is mainly on MSPs, the development of constitutive mechanical models in the absence of any aggressive agents is covered briefly as a precursor, while those focused on MSPs have been covered in detail. Amorphous and semicrystalline polymers differ in failure mechanisms both in dry conditions and in the presence of a fluid. This review is focused on amorphous polymers because in semicrystalline polymers (SCPs) too, it has been well-established that the amorphous phase almost entirely accounts for the MSP including both ESC and MSC. There is a large body of literature on polyethylene, polystyrene, polyamide and other SCPs showing that ESC is associated with interlamellar deformation in the amorphous phase between the crystalline lamellae [3,9,80,81]. In the following description, a brief description on SCPs is presented first, following which the constitutive models for amorphous polymers have been discussed, beginning with rubber elasticity and leading up to those that can span the glass transition across frequency, temperature, and the fluid fraction, including those constitutive models with a molecular basis. The final section is dedicated to viscoelastic fracture mechanics approaches most suited for MSP.

### 4.1. Semicrystalline Polymers (SCPs)

Most models for semicrystalline polymers represent them as a composite of amorphous and crystalline regions. It has long been established that the entanglements and molecular intercrystalline connections in the interfacial amorphous phase, such as tie molecules, act as stress transmitters (STs) and play a crucial role in determining the nature of mechanical failure following the fracture of crystalline lamellae into small blocks [82,83,84,85,86,87]. Following crystal fragmentation, the elongation of the STs allows ductile failure of SCPs such as polyethylene (PE) and polypropylene. The brittle fracture stress for PE has been found to increase with tie molecule content, forcing a ductile failure. Cho [85] unveiled the individual roles of crystallinity, crystal thickness and tie molecule content in governing the relative magnitudes of brittle fracture stress and ductile yield stress. For a variety of SCPs that undergo ESC, local plasticization via the aggressive agent behind the crack tip in the so-called process zone or failure zone facilitates tie molecule disentanglement and disrupts the micromechanism of deformation guided by the STs [83,84,88,89,90], leading to brittle failure at relatively low stress.

Although there are phenomenological constitutive models that can make accurate predictions of the macroscopic mechanical behavior of semicrystalline polymers, when the end goal is to characterize phenomena such as ESC or MSC and predict them for specific polymer–fluid combinations, micromechanical models that explicitly incorporate the underlying molecular and mesoscale mechanisms are far more attractive. Constitutive models that explicitly incorporate the mechanical damage caused by pullout of tie molecules and fracture of crystalline lamellae have been developed recently. The influence of void initiation, coalescence and stabilization in the process zone of SCPs can be determined from molecular dynamics simulations [91] and used to generate appropriate traction separation laws to use in a meso-scale theory. The traction separation laws can also be generated using suitable micromechanical constitutive models for SCPs. A review of single-, two-, and three-phase micromechanical models, which discusses the evolution and incorporation of experimentally observed deformation and damage mechanisms as well as global–local deformation relationships in SCPs, has been compiled by Mirkhalaf and Vadizadeh [92] and is not repeated here. For the reasons introduced previously, the remainder of Section 4 is primarily dedicated to amorphous polymers.

The primary characteristics required in any constitutive model for an MSP are as follows:

(a) Validity across the glass transition: To capture the viscoelastic, viscoplastic and fracture behavior across the glass transition as a function of mechanical stress, temperature, and crucially, the fluid concentration.
(b) Volume change independence: The polymer–fluid system volume is not equal to the sum of the volumes of the dry polymer and the solvent during the dry-to-wet transition, in contrast to the case of a hydrogel whose free volume is already saturated.
(c) Fluid concentration-dependent properties: Both viscous and elastic properties evolve strongly across the glass transition during the dry-to-wet transition of the polymer. The model should incorporate the effect of fluid sorption on viscoplasticity, damage and fracture. The model should also incorporate the appropriate plastic behavior to capture the irreversible part of MSC associated with bound solvent.
(d) Built from a molecular basis so that the model parameters can be traced back to the fundamental physical and chemical descriptors of the polymer and fluid.
(e) Fit into a computational framework capable of handling both fracture and large deformation to be able to seamlessly transition from an ESC-type brittle fracture to an ESY/MSCR-type ductile failure.

Constitutive models from the literature have been reviewed considering these characteristics, beginning with statistical mechanics-based models beyond the glass transition temperature, leading up to molecular-based theories, which incorporate several of the required characteristics for MSP.

### 4.2. Constitutive Models Spanning the Glass Transition Across Temperatures

As MSP invariably spans the glass transition, constitutive models for MSP must be able to do so. Phenomenological viscoelastic constitutive models such as the standard linear solid can span the glass transition across frequency. Using the time–temperature superposition (TTS), the model can be extended to obtain the temperature dependence of the mechanical behavior. The TTS principle assumes that the various relaxation modes of a polymer chain experience identical friction, and hence the relaxational dynamics of those modes couple with each other with the same temperature dependence [93]. However, it has been reported that the TTS principle can break down near the glass transition [94,95,96,97,98,99,100,101,102,103]. If the TTS principle is experimentally shown to hold across the glass transition, it can be exploited to capture the temperature dependence of viscoelastic properties of the polymer across the glass transition and used to greatly reduce the number of required experiments and the complexity of the constitutive model. The TTS principle has been extended to certain polymers for, e.g., polypropylene–ethylene copolymer [104], and asphalt [105]; the same principles as for viscoelasticity have been shown to apply. However, for both viscoelasticity and viscoplasticity, the validity of the TTS principle must be investigated before applying it.

To simulate processes operating around the glass transition, e.g., hot drawing and thermoforming, Buckley and Jones [66] formulated a constitutive model based on the decomposition of free energies into two components based on bond stretching and configurational entropy respectively. This was the first to span the glass transition, as well as incorporate, via the Eyring theory, the feature that the same processes or mechanisms controlling the viscosity also controlled the plastic yield. More recently, other constitutive models for amorphous polymers that span the glass transition have been developed [106,107]. In the work by Dupaix and Boyce [108], a parallel-structured constitutive model was developed to describe the finite-strain behavior of amorphous polymers across the glass transition for polyethylene terephthalate (PET) and polyethylene terephthalate glycol (PETG). The model combines intermolecular interactions and network resistance in parallel and incorporates the physically based and experimentally validated temperature dependence of viscoelastic model parameters and plastic strain rates. A similar constitutive law spanning the glass transition across temperature was proposed by Srivastava [107]. The model separately considered each micromechanism of deformation and proposed rules for the evolution of viscoplastic model parameters with temperature for Zeonex, Polycarbonate and PMMA, and was able to simulate phenomena such as plane strain forging, blow-forming, and micron-scale hot embossing. Das [106] proposed a two-temperature constitutive law based on configurational and kinetic–vibrational temperatures, corresponding to the entropic and energetic contributions of the underlying deformation mechanisms, and demonstrated the model’s ability to capture decreasing yield stress with temperature. Other constitutive models that span the glass transition through temperature include Chen and Schweizer [109] and Liu et al. [110]. The separation of deformation into individual molecular deformation and relaxation processes is an important feature of the above models, which can be utilized in modeling the interaction of polymers with fluids, since only certain micromechanisms may be affected by fluid sorption.

Although the models discussed in this section successfully span the glass transition across temperature, and incorporate molecular-scale deformation mechanisms, to model MSP, it is required that the constitutive model also captures the glass transition across solvent concentrations. The incorporation of both equilibrium and non-equilibrium thermodynamics of polymer–solvent interactions is highly essential since the prediction of MSP is desired for specific polymer and fluid combinations. The following Section 4.3,Section 4.4,Section 4.5,Section 4.6 address these aspects.

### 4.3. Poromechanical Constitutive Models

The development of constitutive material models for MSP is preceded by the development of poromechanical constitutive laws for hydrogels where the polymer is already saturated with fluid. The study of coupled fluid transport and solid deformation in poromechanics originated from classical theories of porous media flow, such as Gibbs’ thermodynamics [111], Biot’s theory of poroelasticity [112,113,114], Coussy’s theory of poromechanics based on continuum mixture theory [115], as well as various micromechanics-based theories [116,117,118], and was later generalized for stress-assisted diffusion frameworks by researchers like Rice and Cleary [119]. However, the above theories were developed largely for application in rock mechanics and represented the fluid in pore spaces as a bulk fluid. The assumption of pore fluid as being equivalent to bulk fluid breaks down when pore sizes approach nanometer and molecular-length scales, particularly for low fluid concentrations and for bound fluid, when the intermolecular forces between fluid molecules in the pore spaces and the solid surface or polymer chains are closer to permanent bonds in solids as opposed to transient bonds in fluids [120]. Poromechanics specifically applicable to microporous media has been developed to address this case [68,69,71,72,121,122,123]. These models utilize the Maxwell relationship to develop a constitutive law where the sorption is a function of both strain and chemical potential. They are however in the realm of reversibility, so while they are excellent at predicting the effect of mechanical stress on sorption, they need to be enhanced to accurately capture the effect of sorption on mechanical behavior. In particular, dissipative processes for viscoelasticity and plastic dissipation need to be included, coupled with sorption, since it is crucial that the constitutive law spans the glass transition to model MSP, especially when the sample transitions from dry to wet conditions.

Poromechanical theories for saturated hydrogels have been developed by several researchers, notably Suo [124,125,126,127]. Hu and Suo [125] developed a comprehensive poroviscoelastic theory to describe the coupled response of elastomeric gels arising from polymer network conformational changes (viscoelasticity) and solvent migration (poroelasticity). However, the viscoelastic component of the free energy was decoupled with the free energy of mixing represented by the chemical potential. As a result, while these poroviscoelastic constitutive models are well-suited to describe the chemo-mechanical behavior of fully saturated hydrogels, they do not account for the effect of the fluid content on the evolution of the polymer’s viscoplastic properties across the glass transition in the dry-to-wet regime. Constitutive models addressing this aspect are discussed in the following section.

### 4.4. Constitutive Models Spanning the Glass Transition Across Fluid Concentrations

Most applications of the Flory–Huggins theory intrinsically assume volume conservation during fluid sorption, which is not generally true for fluid diffusion into a dry polymer, specifically for the bound part of the fluid. Consequently, as pointed out by Govindjee [128], while mixing, the volume conservation rule that necessitates that gel is incompressible and any change in volume is equal to the volume of sorbed water is only true for gels in saturated equilibrium or when the sorbed fluid is stored in a free rather than bound form, but certainly not in the case of dry polymer absorbing fluid. Constitutive models incorporating volume change independence between polymer and fluid, and accounting for the change in mechanical properties due to fluid-induced glass transition, were addressed by Govindjee [128] in his constitutive model to describe Case II coupled stress diffusion, and by Weitsman [128,129,130]. While their models are well-suited to describe MSP, they have unfortunately not been utilized for this purpose.

Kulasinski [131] developed a multiscale poromechanical modeling framework by bridging atomistic molecular dynamics simulations with continuum-scale constitutive modeling, while accounting for all the structural hierarchies in wood. A poroviscoelastic constitutive model that captures the coupling between moisture diffusion, mechanical weakening, and swelling was upscaled from molecular dynamics simulations. The moisture dependence of the material parameters was investigated using both molecular dynamics as well as physical experiments, and the stress dependence of the chemical potential and hence the adsorption isotherm was established through the model parameters of their constitutive model. As a result, this naturally models the dependence of sorption on mechanical stress. However, their approach did not include viscoplasticity, which is crucial for capturing the irreversible part of MSC both from the bound solvent and from mechanical plasticity. The complexity of the stress dependence of chemical potential in their model is strongly in part due to the multiple structural hierarchies in wood structures [132]. It is possible that for sparsely crosslinked linear polymers, the dependence of the reversible component of MSC is much simpler, e.g., as proposed by Govindjee [128]. For the simplest cases, it is likely possible to model the equilibrium thermodynamics of polymer–solvent mixing based on statistical mechanics along the lines of Flory [133] with minimal experimentation. However, for more complex polymers such as cellulose-based nanostructures, a multi-scale investigation from the molecular scale [68,69,71,72,123], supplemented by solvent sweep experiments, is strongly justified.

**Figure 7:**
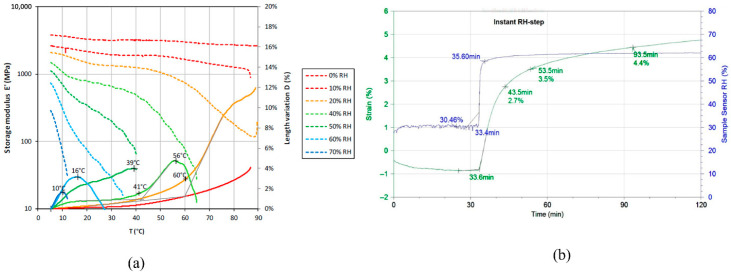
(**a**) Humidity–temperature sweep for storage modulus and (**b**) mechano-sorptive creep strain, reproduced from Bonnaillie and Tomasula [134].

Experiments revealing the dependence of dynamical mechanical properties on the solvent concentration, temperature and frequency, for example, temperature–solvent sweep experiments using DMA, would greatly aid in the understanding of MSP. Although there is a large body of data available comparing the isochronal temperature sweeps of dry and saturated polymers, there are only a few examples of solvent-sweep data in the literature. Bonnaillie and Tomalusa [134] performed a humidity–temperature sweep using dynamic mechanical analysis (DMA) on moisture-sensitive edible casein films: part of their results has been reproduced in Figure 7.

To span ESC-type behavior, it is also important that the constitutive models incorporate some form of damage [65,135] or fracture mechanics, which is discussed in the next section.

### 4.5. Visco-Elastoplastic Fracture Mechanics and Continuum Damage Mechanics for MSP

As opposed to MSC or MSCR, the focus in ESC is to understand the slow crack propagation in the presence of the ESC agent, and fracture mechanics are the most appropriate tool for this purpose. The field of viscoelastic fracture mechanics has been developed to capture unique features of fracture in viscoelastic materials: the rate dependency of fracture toughness, the existence of discontinuous and continuous crack propagation modes [136,137], rate-dependent variations in the damage process zone size and characteristics, and the rate-dependent nature of the crack tip (increasing sharpness with crack speed). However, only Linear Elastic Fracture Mechanics (LEFM) has been primarily used to study ESC [138,139,140,141], although the variation in the stress intensity factor K_I_ has been analyzed with respect to crack tip velocity [142].

Crack initiation and propagation in viscoelastic materials are preceded by the following damage mechanisms: void nucleation, fibrillation and drawing, leading to void coalescence, followed by fibril rupture, which occur in a localized region immediately behind the crack tip, termed a process zone (PZ). Rupture by chain scission or disentanglement leads to the formation of new cracked surfaces and extension of the crack. Crack growth is governed by energy dissipation via plastic deformation and damage in the PZ as well as viscoelastic dissipation in the bulk of the polymer. In semicrystalline polymers, the damage process in the process zone initiates with interlamellar deformations in the amorphous phase [143]. In addition, plastic dissipation through localized sliding in the amorphous and crystalline regions also plays a role in the process zone of semicrystalline polymers and is revealed as shear banding on the polymer surface. The size of the PZ depends on the constitutive behavior of the material [144] and can vary from a few nanometers for a perfect elastomer to a few hundred microns for viscoelastic polymers. The region behind the crack tip is described as a “viscoelastic trumpet” by de Gennes [145] consisting of a glassy region surrounding the process zone, which in turn is surrounded by a viscoelastic zone, and finally, by a rubbery region due to the spatial variation in loading rate around the crack. By comparing model predictions of PZ sizes against mechanoluminescence experiments, Barthel and co-workers [144] showed that linear viscoelasticity when calibrated by dynamic mechanical analysis is indeed applicable to the modeling of fracture propagation in polymers with a significant viscous contribution to the mechanical behavior. However, application of linear viscoelasticity to describe fracture propagation in elastomers for typical crack velocities predicts unrealistically small process zone sizes due to their extremely high glass transition frequencies.

Analytical Models: Analytical models for Mode I cracking have been used to derive highly insightful viscoelastic fracture mechanics theories. Schapery’s analytical model for viscoelastic fracture [146,147] utilizing the generalized correspondence principle predicts the crack opening displacement (COD), process zone size, as well as variation in fracture energy with crack propagation rate. The fracture energy follows the experimentally observed sigmoidal-shaped curve [148,149] with respect to the velocity of crack propagation: several viscoelastic fracture mechanics constitutive laws can predict this trend [144,145,147]. This trend is non-trivial and is governed by the variation in viscoelastic properties of the material with frequency. It can be understood based on Schapery’s application of the correspondence principle to viscoelastic fracture mechanics. In fact, the transition from an ESC-type brittle fracture to an MSC type of large deformation ductile failure can be understood based on Schapery’s viscoelastic fracture mechanics theory, specifically Equation (53) of [147]. Schapery’s theory predicts the existence of a lower-bound stress intensity factor K_Ie_, below which fracture does not propagate, and an upper-bound stress intensity factor K_Ig_, above which fracture propagates spontaneously, both of which have been experimentally verified [142]. In the case of a compatible solvent, although the solvent would cause the fracture energy Γ to decrease, bulk plasticization is expected to cause the stress intensity factor K_I_ at the crack tip to decrease rapidly to less than K_Ie_, causing crack blunting and large deformation. On the other hand, in the case of a weak-to-poor solvent, or low solvent concentration [60], localized plasticization, as qualitatively proposed [3,35,150], would only result in a negligible reduction in K_I_, whereas the reduction in fracture energy would be substantial, resulting in a brittle ESC type of failure. Moreover, Schapery’s theory, specifically Equation (54) of [147], also predicts the remarkable increase in crack propagation rate in an ESC agent as opposed to air, by virtue of the significant decrease in the fracture energy Γ. The fracture energy Γ can be calibrated using either experiments or modeling at smaller scales. It is indeed unfortunate that Schapery’s analytical model has not been utilized to study MSP.

J-Integral for Viscoelastic Fracture Mechanics: The stress in a viscoplastic solid cannot be expressed as a derivative of the Helmholtz free energy with respect to the elastic component of the strain. Consequently, as pointed out by Rice [151], the J-integral is not path-independent and is not representative of the energy release rate with respect to crack growth, as in the case of metal plasticity. While the crack-driving forces required to model fracture propagation can be obtained using the J-integral for time-independent plasticity, they cannot be applied in the same manner to time- and rate-dependent plasticity and fracture. However, both Griffith’s theory of LEFM and Rice’s J-integral are based on calculating the energy release rate with respect to crack growth using the difference between external work and internal energy. This concept can still be utilized in the case of viscoplasticity. Configurational thermodynamic forces have been effectively used to drive crack propagation in viscoplastic fracture models such as the crack layer theory by Chudnovksy [137]. In crack layer theory, the crack and process zones (PZs) are treated as a coupled system, with their evolution driven by configurational thermodynamic forces. Chudnovsky’s crack layer theory has successfully been used to reproduce and explain the conditions that lead to continuous versus discontinuous crack growth [152]. Similarly, the Eshelby energy-momentum transfer tensor can be used to derive the required configurational thermodynamic crack-driving forces for visco-elastoplastic materials [153,154,155]. Schapery’s work on the generalized J-integral [156] extends the applicability of his theory to large deformation viscoplastic behavior.

Numerical Models: Coupled large deformation problems for hydrogels with a moving diffusion front but without fracture have been successfully formulated [157,158,159,160,161] and solved also as MSC problems [32,131,162] using finite element or finite difference methods for example. Semi-phenomenological constitutive laws developed by Singh and Misra for methacrylate-based dentin adhesives [50,65,135,163] and by Reichel and Kaliske [31,32] for wood have successfully employed continuum damage mechanics to capture primary, secondary, and tertiary aspects of creep, including the mechano-sorptive effect on damage. Despite the successful implementation of damage mechanics to model stress corrosion cracking [164,165,166,167], it has not been utilized to study ESC.

Simulation of viscoelastic fracture mechanics of MSP for arbitrary geometries and boundary conditions involves simultaneous handling of the sharp or diffuse swelling front with large deformations behind the crack tip in addition to fracture propagation. As explained in Section 3, the stress concentration behind the crack tip will induce rapid diffusion and localized swelling, thus reducing relaxation times. However, the high strain rates behind the crack tip may cause the loading rate to exceed the relaxation rate; therefore, careful analysis is required to characterize the constitutive behavior in this region, especially as the PZ size reduces. To regularize the problem, accurately represent chemomechanical evolution and avoid mesh-dependent solutions, a non-local formulation is required. Numerical methods that are most promising to address these challenges include the phase field method (PFM) [161,168,169,170,170,171,172,173,174,175,176], peridynamics [177,178,178] and strain gradient-based damage modeling. It is noteworthy that both peridynamics and PFM have been used for modeling stress corrosion cracking, but not ESC or MSC. PFM is well-suited for problems involving solvent penetration into the polymer since they can regularize a moving boundary into a diffuse layer of finite thickness. However, when damage is represented by a phase field variable, the physical meaning of Neumann boundary conditions is unclear. Similarly, peridynamics, like PFM, are well-equipped to handle moving boundary problems but suffer from unphysical boundary conditions. Placidi, Barchiesi and Misra [179] developed generalized continuum damage models where the damage variable is a function of the strain gradient, whereby the regularization of stresses at the crack tip is a consequence of the physics of the problem rather than a numerical coercion. Strain-gradient theory for viscoelastic fracture shows significant differences compared to strain gradient elasticity [180,180]. Damage gradient and strain gradient models for polymers where the length scale is physically linked to the microstructure in the PZ through statistical mechanics have also been developed [181,182,183]. Deformation mapping connecting the first- and higher-order continuum kinematics to the polymer chain stretching, disentanglement and scission in the PZ, e.g., as done by Mousavi [183], is crucial to ensure a wide range of predictive ability of the model, especially across the glass transition. Furthermore, it is of utmost importance that the intrinsic length scale parameters that the approach uses should be calibrated to match the experimentally observed sizes of the PZ, the thickness of the diffusion front, and the volume it encompasses.

In all non-local models for polymers, the model parameters are functions of multiple length scales including the PZ size, the persistence length, the contour length, as well as others, which can complicate their experimental identification [184,185]. Nevertheless, it appears that if the solvent concentration is included as a constitutive variable, the strain gradient method is well-suited for MSP, provided that the model parameters can be linked to the underlying structure through statistical mechanics, experiments, or molecular modeling.

### 4.6. Mesoscale Approaches with a Molecular Basis for Viscoelasticity, Viscoplasticity and Fracture

The first constitutive model for polymer mechanics to be derived directly from statistical mechanics is that of rubbery elasticity by Flory in 1943 [133]. The modeling of mechano-sorptive processes necessarily involves the effect of polymer–solvent interaction on the viscoelastic behavior of the polymer and consequently requires the constitutive model to span the glass transition. Constitutive models spanning the glass transition due to fluid sorption must be able to separate the free energy change into energetic and entropic contributions [66,106,186]. As demonstrated by Flory and others, statistical mechanics can be used to bridge the molecular and macroscopic descriptions of polymer mechanics involving polymer–solvent interactions without explicitly using molecular simulations. However, Flory’s method of computing free energy of mixing is ideally suited only to rubbery regimes where the polymer chains are already completely solvated, and the total free energy can be accurately computed using the Boltzmann entropy since the time scale of loading is several orders of magnitude larger than the polymer dynamics. This method cannot be extended to the glassy and viscoelastic regimes where internal energy changes are significant, and the time scales of polymer chain dynamics can extend from picoseconds to days.

Consequently, several mesoscale theories of viscoelasticity applicable to network polymers or crosslinked polymers have been developed. The two critical components of modeling are as follows:

(a) The molecular-to-continuum kinematics bridging between the macroscopic deformation and the polymer chain segment dynamics and stretching;
(b) Convection–diffusion equation representing polymer dynamics;
(c) Averaging to determine model parameters from lower-scale simulations for, e.g., all-atom MD simulations.

Theories based on molecular descriptions undoubtedly have the advantage of a physically based bottom-up prediction of mechanical properties, allowing multi-scale, multi-physics validation of constitutive laws. For example, the Flory–Rehner theory [133] has been highly successful in describing the behavior of rubbers and has been used to develop poromechanical theories of hydrogels, particularly by Suo and co-researchers [124,125,126,127]. Poromechanical theories leveraging Maxwell’s relationships have been developed where the sorption isotherm is dependent on chemical potential as well as strain [68,69,72,121,123,131]. Such theories are more general because this dependence is obtained from experiments or molecular simulations without any pre-assumptions. When combined with a suitable dissipation potential to account for viscous and plastic effects, these theories are suitable for MSP.

Similarly, there are several molecular theories of viscoelasticity applicable to network polymers or crosslinked polymers, but they are limited to entropic forces on the polymer chains. The internal energy changes due to bond stretching and bending are only considered at large deformations. Therefore, these theories, although useful in the viscoelastic regime, are not extendable to the glassy regime. Examples include the Sticky Rouse model [187] for network polymers, which derives the macroscopic viscoelasticity of polymers from the diffusion of the crosslink centers as well as individual chain segments via the Langevin equation or the Smoluchowski equation, and the Kremer and Grest model [188] for entangled linear polymer melts. The transient network theory [189,190,191,192] connects the time-evolving stress tensor to macroscopic viscoelasticity in the Maxwell or general Boltzmann superposition form via the chain distribution tensor as the molecular-to-macro bridging kinematic variable. The Transient Network Theory was introduced by Green and Tobolsky [193,194], and further developed by Tanaka and Edwards [195] and later by Vernerey [189]. In TNT, viscoelasticity is a consequence of the continuous dissociation and association kinetics of transient interactions between polymer chains. Vernerey introduced the chain distribution tensor in TNT to connect the macroscopic deformation gradient to the evolution of the polymer chain end-to-end vector. Subsequently, the free energy including viscoelastic dissipation and stress was derived in terms of the chain distribution tensor. Under conditions where the dissociation and association times of the reversible bonds are orders-of-magnitude smaller than the time scale of loading, these models reduce to Flory’s rubbery elasticity. On the other hand, when the dissociation and association times of the reversible bonds are orders-of-magnitude larger than the time scale of loading, these models reduce to linear elasticity corresponding to glassy behavior. When the bond dissociation and association times are comparable to the time scale of loading, viscoelasticity becomes significant. However, slow relaxation modes such as diffusion of crosslink centers need to be explicitly incorporated. The Brachiation model [196] employs a memory kernel that links the single chain dynamics to the network viscoelasticity, which enables it to predict the viscoelastic behavior in high-, intermediate- and low-frequency regimes with a different set of molecular parameters governing the viscoelasticity in each regime. While the drag force dominates the short time and length scale behavior, the binding rates and number of associating units control the behavior in the low-frequency regime. It is possible for other models such as the slip link model [197,198], the standard Rouse model, the Doi–Edwards model, and others to incorporate entanglement constraints and finite extensibility and adapt to crosslinked networks. Coarse-grained molecular dynamics [199,200,201] can also be used to model viscoelastic behavior from polymer dynamics. However, there is a paucity of molecular-level theories that can span the glass transition from the glassy regime, through viscoelasticity to the rubbery regime.

Since molecular-scale modeling of MSP requires accurate representation of polymer–fluid interactions at the atomic level, especially hydrogen bonds and specific chemical affinities, it is extremely important to calibrate the model parameters of any mesoscale model using atomistic simulations for, e.g., molecular dynamics. For example, the friction coefficients, bond strength, association lifetime, as well as entanglement equilibration time in an SRM can be calibrated using an MD simulation.

As compared to molecular-based mesoscale approaches to model linear viscoelasticity, molecular-based mesoscale approaches to model fracture and damage are in a more nascent stage. The Lake–Thomas model and adjusted versions [202,203,204] represent efforts in the rubbery regime to connect fracture energy to molecular mechanisms viz. chain stretching followed by scission. TNT has been applied to an idealized polymer to model fracture propagation [205] and damage mechanics [206,207] with the inclusion of viscoelastic dissipation. A closed form expression for fracture toughness was derived for the case of viscoelasticity dependence on bond dynamics only, while a damage parameter was proposed based on the evolution in the number of ruptured chains and extension limits for a single chain. It is acknowledged that the dependence may be much more complicated, and a closed-form solution may be unavailable for polymer–solvent interactions. This is particularly true since the polymer mechanics can span the glass transition with a change in fluid fraction. The quasicontinuum approach developed by Tadmor [208] has been implemented to study fractures of various types of polymer networks [209]. The region behind the crack tip is modeled at the molecular scale using criteria such as rate-dependent bond breakage and formation of end bonds and crosslinkers and viscous drag on the deforming polymer network. The bond breakage was modeled using transition state theory. The model can capture both discontinuous and continuous crack propagation modes for idealized viscoelastic networks with both permanent and reversible crosslinks.

There are a few theories that span the glass transition, e.g., the Mode Coupling Theory (MCT) [210,211], the Gibbs–DiMarzio theory [212], and the disorder-assisted theory of Lappala et al., which incorporate the non-affine reduction in shear modulus arising from structural disorder [213,214]. However, the particle-scale quantities, such as intermediate scattering function or connectivity, used to identify the glass transition do not have immediately apparent continuum mechanics analogs. Examples of molecular-based continuum theories that span the glass transition include TNT and the Tube-Junction model by Simon and Ploehn [215]. The Tube-Junction model captures the dependence of the glass transition on temperature, frequency, as well as plasticizer fraction by accounting for cohesive, frictional and entropic forces on the polymer network.

**Figure 8:**
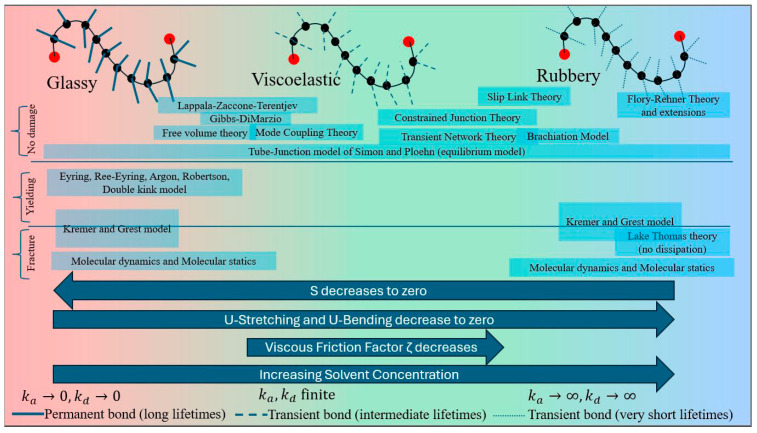
Representation of several modeling approaches showing their applicability across glassy to rubbery regimes (horizontal direction) and across the elastic regime and beyond to yielding and fracture (vertical direction).

There have been only a few instances where either ESC or MSC has been studied at the molecular level. Kavyani [91] used molecular dynamics simulations to study the effects of an ESC fluid on HDPE in terms of increased void number and volume. Alperstein [216] investigated the behavior of polycarbonate in the presence of toluene, water and butadiene using density functional theory and noted that simultaneous reduction in the solubility parameter and increase in free volume was responsible for ESC. Navi et al. [217] found that the application of a shear stress while water diffused out of a supercell of crystalline cellulose layers enhanced deformation rates as in MSC. Molecular-scale observations confirmed that both intra- and inter-molecular hydrogen bonds in the crystalline phase remained unaffected, while suggesting that the presence of stress delays the reformation of the hydrogen bond, leading to enhanced deformation. Stevanic and Salmen [29], using dynamic Fourier Transform (FTIR) Infrared spectroscopy of deformation of cellulose, found an increase in the dissociation of hydrogen bonds under the application of tensile stress, as well as a reduction in the force constants of intramolecular hydrogen bonds. Since the time scales of viscoelastic deformation and the spatial scales of MSP in general are inaccessible to all-atom molecular dynamics, the information from such results cannot be directly used to calibrate material models for MSP. Molecular models must be bridged to meso-scale models and eventually to macro-scale models to predict MSP at relevant scales of application. Figure 8 shows a representation of the various spatial and temporal scales along glassy to rubbery regimes along with the modeling approaches and their applicable ranges. The range across which a model is applicable has been represented by the corresponding textboxes in cyan.

There is currently no molecular-level continuum theory that can span all the regimes while describing the mechanical behavior from small to large strains, yielding and fracture. Owing to limitations in the time and length scale accessible to each approach, a multi-scale model for MSP would need to transition between various regimes according to the local material composition and deformation. In consistency with the De Gennes viscoelastic trumpet [145] around the crack tip, molecular simulations focus on the zone closest to the crack tip where the polymer responds as a glassy solid. In any case, only the high-frequency behavior is accessible to all-atom molecular simulations: they focus on cavitation and crazing at crack tips [218,219,220] and can be coupled with mesoscale simulations of viscoelastic fracture propagation [137,146,156,221]. The most promising molecular based models that appear to be extendable in a straightforward manner to model MSP are the Tube-Junction theory of Simon and Ploehn and the Transient Network Theory proposed by Vernerey.

## 5. Role of MSP in the Functionality and Synthesis of Biomimetic Materials

Dentin Adhesives and Other Dental Materials: The oral cavity has fluctuating moisture contents, temperatures, and stresses, thus making it a worst-case scenario for MSP including MSCR and ESC. In the wet bonding technique, the dentin is kept hydrated to support the collagen matrix of the demineralized layer. As a result, adhesives for class II composite dental restorations are required to diffuse into the spaces along the collagen fibrils and are necessarily partially hydrophilic. In the polymerized state, they retain a degree of hydrophilicity, and are therefore highly susceptible to MSCR [222]. PMMA, which is used to manufacture the base of dentures due to its aesthetics, cost-effectiveness and biocompatibility, is highly susceptible to ESC [60,169,223,224,225,226,227]. Incorporating resistance to failure by MSP into dental materials is thus a crucial aspect of their design.

Tendons and Cartilage: The biomechanical functionality of several natural tissues, e.g., tendons and cartilage, involves MSP. Poroelasticity of cartilage and other tissues is primarily controlled by free water while viscoelasticity is controlled by bound water [228,229,230,231]. Accordingly, controlling the proportions of bound and free water in unloaded and loaded conditions has been identified as a key parameter in mimicking natural extracellular matrix (ECM), e.g., using hydrogels, especially for replicating poroviscoelastic properties [232,233]. It has been shown that inadequately hydrated tendons undergo irreversible deformation like MSCR under simultaneous tension and moisture flux [234,235,236]. D-period disruption and unravelling of crimp patterns have been observed [237]. The replication of these properties is also a crucial aspect of developing biomaterials for annulus fibrosus rupture repair [238]. Results from successful treatment protocols for tendinopathy have also used stress relaxation for healing and applied bound and free water contents as part of a set of benchmarks to evaluate tissue recovery [239]. Thus, a deeper understanding of MSP in native and biomimetic materials using the theoretical arguments and experimental protocols presented in this review is expected to significantly aid the development of synthetic biomimetic materials for biomechanical applications.

Synthetic Scaffolds: Waterborne biodegradable polyurethanes have been successfully explored to simultaneously mimic the hydration state, low modulus, and structural stability of hydrogel mimics for the EMC of central nervous system soft tissue [240]. In the synthesis, structural stability was achieved after several loading–unloading and saturation cycles. However, the explicit contributions of bound and free water for elastic and poroviscoelastic properties were not quantitatively leveraged. In previous work, we have shown the dramatic reduction in modulus with free water [49]. The discussion presented in this review on MSP is expected to significantly enhance the controllability and predictability of the mechanical behavior of synthesized scaffolds in a wet environment. It is also similarly useful to guide the simultaneous achievement of tunable mechanical properties, controlled swelling, dissolution behavior, and sustained release in the development of wound dressings [241]. Several other biomimetic materials rely on bound water for functionality, e.g., biomimetic bone scaffolds, aquaporin-based membranes, hygro-responsive textiles, zwitterionic hydrogels, and artificial cartilage.

Spider silk: Spider silk has generated high research interest due to its outstanding mechanical properties and toughness. It is formed by molecular alignment during web spinning, which is achieved by simultaneous deformation and loss of water. Supercontraction of spider silk is a phenomenon where this process is essentially reversed, resulting in loss of the oriented structure between crystalline regions [14,15,17]. During supercontraction, the interaction between the pre-stress in the “frozen” glassy state and the moisture influx is by definition MSP. Although it is recognized that the synthesized glassy state is not a ground state [13], the phenomenon is yet to be analyzed from the perspective presented in this review. Peptide–cellulose reactions have been used to tune water sensitivity and mechanical performance in biomimetic analogs of spider silk [242]. This review is expected to strongly inform such design.

Mutable Collagenous Tissue (MCT): In MCT found in echinoderms like sea-cucumbers [12], the tissue stiffens and softens by increasing or decreasing chemical crosslinks modulated by tensilin or softenin. The simultaneous prestress and fluid flow make this MSP. Several sea-cucumber-inspired smart hydrogels [12] have been developed, which are responsive to various types of stimuli, including stress, light, and chemical changes. The concepts presented in this review can be leveraged to pre-condition the hydrogels to specific proportions of bound and free water, to tune the resulting viscoelastic properties.

Synthesis of Biomimetic Materials: Solvent vapor annealing assisted by soft shear (SVA-SS) is a method that leverages the synergistic effects of solvent-induced mobility and mechanical stress to aid the alignment of nanodomains in block polymer thin films (BCPs) [19,21,22]. BCPs have been demonstrated to serve as a general platform for patterning of supramolecular structures that are known to assemble at solid–liquid interfaces. They have been used to prepare templates for biomimetic mineral synthesis [243]. Although the terminology used in SVA-SS does not mention MSP, the underlying mechanism shows substantial overlap with MSP. Therefore, the insights into MSP presented in this review can be utilized to guide self-assembly in BCPs from a more predictable standpoint. For further optimizing such self-assembly as well as predicting yet-undiscovered exotic programmable material behavior incorporating MSP, a spiraling Kuhn paradigm, such as the one designed by dell’Isola and Misra for metamaterials [244], can be utilized.

## 6. Conclusions

The following points, important to the experimental, theoretical, and computational investigation of MSP, most of which have been explicitly identified for the first time, have been discussed in this review:

• For the first time, several seemingly disparate mechano-sorptive phenomena from a variety of applications have been brought together under a single review. The interrelationship between several terminologies used in the literature, including MSC, MSCR, ESY, SEDS, and ESC, has been explained.
• MSCR and ESC have been identified as two extremes of the same underlying phenomenon with supporting evidence from a large body of studies from a variety of fields. Several experimental results that show responses resembling ESC and MSCR/ESY for the same polymer for different solvents or different sorption of the same solvent have been reproduced here to justify this conclusion.
• For the first time, it has been hypothesized that the reversible parts of MSC, ESY or SEDS can be explained by the equilibrium swelling of polymer networks. Only the irreversible part of the MSC necessitates the rate of solvent sorption as a constitutive variable. Irreversibility can also arise from plastic yielding. Once proven, this hypothesis is expected to greatly simplify constitutive modeling of MSP.
• A comprehensive review of the range of existing constitutive models in the glassy–viscoelastic–rubbery regime and yield-fracture regime for the study of MSP has been presented with their range of applicability across the entire spectrum of deformation and failure associated with MSP. Key features required in a constitutive model for MSP have been identified and a library of constitutive models available has been grouped accordingly. Experiments and molecular-level methods that can be used to characterize MSP and calibrate suitable material models have also been presented. In particular, the importance of solvent-sweep experiments for MSP characterization has been highlighted. The Tube-Junction theory of Simon and Ploehn and the Transient Network Theory proposed by Vernerey have been identified as the most promising constitutive models with a molecular basis to describe MSP.
• Schapery’s theory of viscoelastic fracture mechanics, with the generalized J-integral, has been identified as a suitable model for MSP involving fracture, although it has not been explicitly used for this purpose. It has been described how this theory can be exploited to generate a macroscopic model, which can transition between a brittle ESC-like failure to a ductile MSCR-like failure.
• Several biological and biomimetic materials for which MSP plays a key role in their functionality have been identified, and the specifics of how MSP regulates their behavior has been explained. The role of MSP in SVA-SS, one of the important methods for the synthesis of biomimetic materials, has been explained.

## Figures and Tables

**Table 1:** Compilation of results that observe both ESC-like and ESY/MSCR-like failure for the same polymer.

Reference	Polymer	Conditions Resulting in ESC-like Failure	Conditions Resulting in ESY/MSCR-like Failure
Ward et al. [74]	Polyethylene	Lower stress, Medium: Igepal	Higher stress, Medium: Igepal
Breen [75,76,77,78]	Polyvinyl chloride (PVC) and chlorinated polyethylene-modified PVC	Medium: n-hexane, n-decane and ethanol vapors	Medium: Benzene and toluene vapor
Arnold [62]	Thermoplastic toughened phenolic resin	Medium: Oil	Medium: Water
Arnold and colleagues [60]	PMMA	Medium: Methanol (short immersion time)	Medium: Methanol (long immersion time)
Arnold and colleagues [60]	PMMA	Water, ethylene glycol, 355TMH (poor solvent compatibility)	Not observed for these solvents
Schilling and colleagues [64]	HDPE	Medium: Arkopal	Medium: Diesel, biodiesel
Hargreaves [23]	PMMA	Medium: Vegetable oil	Medium: ethanol, sodium citrate solution, and hydrochloric acid
Al-Saidi [79]	Polycarbonate	Medium: Ethylene glycol monomethyl ether (good solvent, surface effects dominate)	Medium: Methanol (good solvent, bulk plasticization dominates)

## Data Availability

No new data were created or analyzed in this study.

## Abbreviations

The following abbreviations are used in this manuscript:

MSP	Mechano-sorptive phenomena
ESC	Environmental stress cracking
MSC	Mechano-sorptive creep
ESY	Environmental stress yielding
MSCR	Mechano-sorptive creep rupture
SEDS	Stress-enhanced diffusion and solubility/swelling
PTFE	Polytetrafluoroethylene (Teflon)
SCP	Semicrystalline polymer
ECM	Extracellular matrix
BCP	Block copolymer thin film
TNT	Transient Network Theory
SRM	Sticky Rouse model
PFM	Phase field method
PZ	Process zone
MCT	Mutable collagenous tissue
SVA-SS	Solvent vapor annealing assisted by soft shear
